# Photoelectric Composite Three-Phase Flow Sensor for Complex Oil and Gas Wells

**DOI:** 10.3390/s26030808

**Published:** 2026-01-26

**Authors:** Qiang Chen, Xueguang Qiao, Tao Chen, Hong Gao, Congcong Li

**Affiliations:** 1 Xi’an Key Laboratory of Optical Fiber Sensing Technology for Underground Resources, School of Physics, Northwest University, Xi’an 710127, China; zycjchenqiang@cnpc.com.cn; 2 Logging Technology Research Institute, China National Logging Corporation, Xi’an 710077, China; chtao@cnpc.com.cn (T.C.); licc.cnlc@cnpc.com.cn (C.L.); 3 College of Science, Xi’an Shiyou University, Xi’an 710065, China; gaohong@xsyu.edu.cn

**Keywords:** complex oil and gas wells, photoelectric composite, three-phase flow sensor, water holdup, gas holdup, flow velocity

## Abstract

Reliable measurement of multiphase flow is fundamental to production evaluation in complex oil and gas wells. However, conventional sensors often suffer from low integration, limited measurement capability, and potential environmental impact. To address these challenges, a photoelectric composite three-phase flow sensor is developed, integrating multiple electrode rings for water holdup and liquid-phase velocity measurement, with dual optical-fiber probes for gas holdup and gas-phase velocity detection. A slip model is further applied to quantify the dependence of slip velocity on liquid holdup based on measured phase rates. Experimental results demonstrate high sensitivity to bubble-flow structures, accurate extraction of gas holdup and phase velocities, and stable full-range water holdup calibration from 0% to 100% at 5 V and 15 V with effective temperature and salinity compensation. And compared with existing technologies, the sensor designed in this paper has the advantages of high integration, a simple structure, multiple measurement parameters, and higher water-holding capacity resolution in low-saturation areas, providing more advanced technical means for conventional profile three-phase flow logging.

## 1. Introduction

Petroleum and natural gas remain among the most essential global energy resources, supporting energy security, industrial production, and economic development [1,2,3]. However, during long-term field exploitation, reservoir pressure gradually declines and oil output decreases, which commonly leads to the adoption of waterflooding for pressure maintenance and recovery enhancement. As flooding progresses, water cut increases and multiphase flow behavior in the wellbore becomes more complex, making production evaluation significantly more difficult [4]. To address this issue, multi-dimensional production logging technologies such as 21 mm annular multi-parameter logging, horizontal well flow imaging and intelligent zonal monitoring are applied to characterize downhole fluid distribution [5]. These technologies are of great significance for stabilizing oil production, regulating water holdup and improving overall operational efficiency and recovery [6,7,8].

The evaluation of multiphase production behavior largely depends on three fundamental downhole parameters, namely water holdup, gas holdup and phase flow rate. Water holdup reflects the degree of water intrusion and remaining oil saturation, and has therefore been measured using capacitance probes [9,10], electromagnetic wave sensors [11], microwave resonators [12], impedance systems [13] and sampling tools [14]. Gas holdup in contrast is more commonly determined using chemical analysis-based techniques [15], which reflects the different sensing mechanism required for dispersed gas phases. Phase flow rate is typically obtained using tracer technology [16], turbine flowmeters [17], ultrasonic transducers [18] and electromagnetic meters [19], enabling quantitative assessment of zonal contribution along a wellbore. Collectively these instruments provide the technical basis for current production logging and support specific measurement of water, gas and flow rate in downhole environments.

Recent work has further improved the performance of independent sensing techniques. For water holdup measurement, Dai et al. [20] developed a 360° dual-helical capacitance sensor and hydrodynamic tests verified reliable measurement across the range of 0 to 100 percent water holdup with sub-percentage resolution in dispersed oil-in-water flow. Wei et al. [11] introduced a coplanar waveguide sensor whose simulated and experimental results confirmed a quasi-linear phase shift response with approximately 3 percent full-scale resolution across the full measurement range. Bai et al. [21] proposed a mesh-structured microwave resonant sensor operating at 1.85 to 2.30 GHz, which showed high sensitivity under high water-cut and low-flow-velocity conditions. Ji et al. [13] further developed a non-contact impedance sensor that avoids polarization and electrochemical corrosion in gas–liquid void fraction measurement. Progress has also been made in gas and flow rate sensing. Yang et al. [14] proposed a variable area electrode capacitance sensor that achieved an average relative water holdup measurement error of approximately 10 percent during upward oil water two-phase flow. Pant et al. [16] applied radioactive tracer technology to obtain residence-time distribution in industrial heavy-oil reactors and successfully identified bypass and dual-path flow. Liu et al. [22] evaluated dual-rotor turbine flowmeter performance through laboratory experiments and cavitation simulations. Shi et al. [18] reported a hybrid electrical and ultrasonic metering system for multiphase flows, and Liu et al. [19] combined electromagnetic and differential pressure sensing for inclined oil water flow and established a holdup-dependent meter-factor correction model.

Complementing electrical and mechanical sensing approaches, optical and photoelectric technologies have emerged as high-fidelity alternatives for multiphase flow diagnostics. By leveraging refractive-index contrasts among gas, oil, and water phases, fiber-optic probes facilitate direct phase discrimination independent of fluid conductivity. For instance, Hao et al. developed a dual-receiver fiber-optic array capable of oil–gas–water holdup measurements with errors within ±10% across diverse flow regimes, validated through ray-tracing sensitivity modeling [23]. Similarly, Kong et al. optimized a fiber-optic array for horizontal gas–liquid stratified flows, achieving gas-holdup errors below 0.1 under low-velocity conditions [24]. Furthermore, Zhang et al. introduced a novel photoelectric approach using fiber Bragg gratings to identify bubble-collision and bounce dynamics, enabling detailed cross-sectional flow characterization [25]. Collectively, these advancements highlight the robustness of optical sensing in complex flow structures and harsh downhole environments, offering superior electrical isolation, miniaturization, and phase selectivity.

Although the above instruments can measure water holdup, gas holdup and phase flow rate with reasonably high accuracy, several limitations remain. These limitations are reflected mainly in the following aspects: (1) the size of instruments restricts further miniaturization for annular production logging tools; (2) complex internal structures reduce the feasibility of highly integrated multi-parameter acquisition; (3) flow rate measurement is often effective only under single-phase or high-velocity conditions and is easily affected by high viscosity crude and debris; (4) gas holdup measurement based on chemical or sampling approaches may cause groundwater contamination, making it inconsistent with modern environmental sustainability requirements.

In response to these limitations and building upon existing sensing methodologies for water holdup, gas holdup, and phase flow rate, this study designs and develops a photoelectric composite three-phase flow sensor. The proposed sensor integrates multiple electrode rings for water holdup and liquid-phase flow rate measurement with dual optical-fiber probes for gas holdup and gas-phase flow rate determination. Experimental results verify that the sensor achieves high measurement accuracy with simplified structure and improved integration. It provides reliable support for simultaneous acquisition of water holdup, gas holdup, and flow rate under complex downhole conditions in oil and gas wells.

## 2. Principle Analysis and Discussion

As discussed in the introduction, the proposed photoelectric composite three-phase flow sensor is designed to measure downhole water holdup, gas holdup, and fluid flow rates. This section therefore explains the underlying measurement principles for these three parameters, providing the basis for the subsequent sensor design and experimental analysis.

### 2.1. Gas Holdup Measurement with Fiber-Optic Probes

Gas holdup measurement using an optical-fiber probe is primarily based on the principle of total internal reflection [26]. Owing to the difference in refractive indices between gas and liquid phases, variations in the intensity of reflected light can be used to identify the phase in contact with the probe and thereby determine the gas holdup in multiphase flows. Specifically, when the probe tip comes into contact with the gas phase, the incident light undergoes total internal reflection at the probe end face, and the photodetector outputs a high-level signal. In contrast, when the probe tip is in contact with the liquid phase, most of the incident light is refracted out at the probe end face, resulting in a low-level output from the photodetector. Gas holdup is then calculated by statistically analyzing the number of high-level signals and their corresponding residence times.

**Figure 1:**
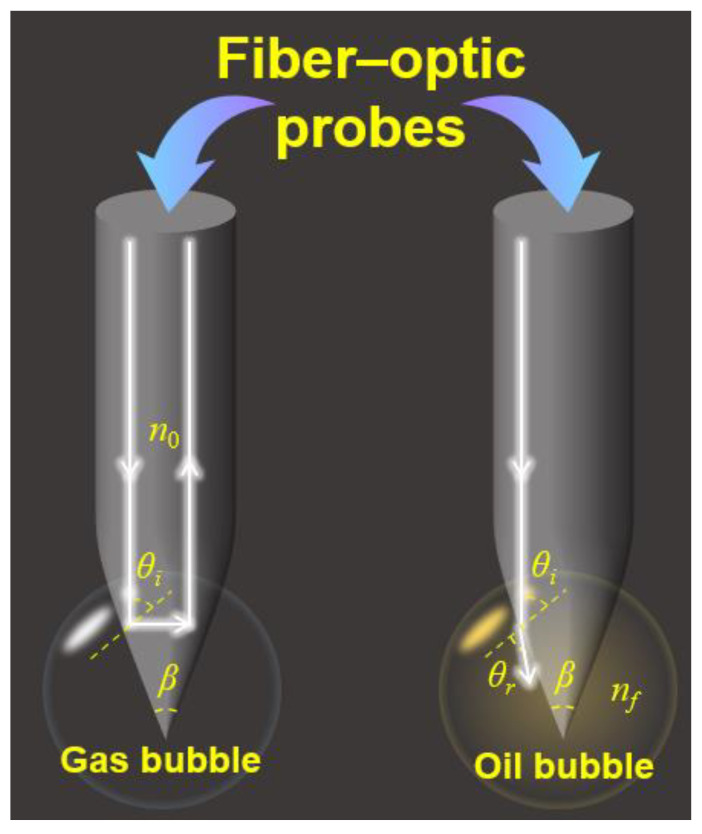
Schematics of gas holdup measurement using fiber-optic probes.

Furthermore, when a light beam is incident on the conical surface of the probe tip, the light path and the associated angles are as shown in Figure 1,The white light represents the light in the bubble, and the yellow light represents the light in the oil bubble. Here, *θ_i_* and *θ_r_* denote the incident and refracted angles, respectively, *n*_0_ is the refractive index of the probe cone region, *n_f_* is the refractive index of the surrounding fluid, and *β* is the apex angle of the probe tip. The value of *β* determines whether the sensor can effectively distinguish between gas and liquid phases. When the incident angle *θ_i_* is 90°, i.e., *β* = 0, the medium in contact with the probe cone surface can be identified as gas, and the incident light undergoes total internal reflection at the cone surface. Under this condition, the critical refractive index *n_rc_* for total internal reflection at the probe cone surface is given by Equation (1).

(1) $$n_{rc}=n_{0}\cos\left(\beta /2\right)$$

Therefore, for the probe to discriminate between gas and liquid phases, the refractive index *n_rc_* determined by the probe apex angle *β* must satisfy the relationship given in Equation (2), where *n_g_* and *n_f_* are the refractive indices of the gas and liquid phases, respectively.

(2) $$n_{g}<n_{rc}<n_{f}$$

From the above analysis, it follows that for an optical-fiber with a given refractive index, appropriate design of the probe tip cone angle can ensure total internal reflection when the surrounding medium is gas, thereby enabling reliable measurement of gas holdup in downhole fluids.

In this study, a quartz optical fiber with a refractive index of 1.5 is selected to fabricate the probe. This fiber exhibits favorable mechanical strength, radiation resistance, and high-temperature tolerance, making it suitable for complex downhole environments. For oil–gas–water three-phase flow under standard conditions, the refractive index of the oil phase is *n_o_* = 1.48–1.50, that of air is *n_g_* = 1.000, and that of the water phase is *n_w_* = 1.333. Consequently, the minimum refractive index of the surrounding fluid is *n_f_* = 1.33, which leads to 1 < *n_rc_* < 1.33. According to Equation (1), this corresponds to an apex angle range of 28° < *β* < 48°. Considering mechanical strength requirements, a probe tip apex angle of 45° is selected for subsequent experiments.

### 2.2. Principle of Capacitance-Based Water Holdup Measurement

**Figure 2:**
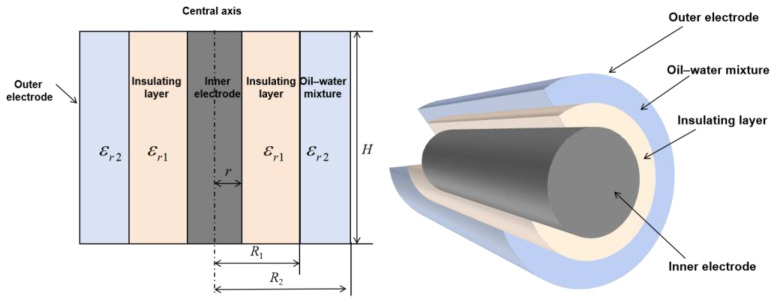
Schematic diagram of coaxial capacitor configuration in cross-sectional and top views. The dotted line is central axis of inner electrode.

Given the significant difference in dielectric constants between oil and water in downhole fluids (under ambient conditions, the relative permittivity of oil is approximately 2.3 while water is approximately 80), capacitance-based methods can be employed for water holdup measurement. The principle can be summarized as follows: when an oil–water mixture flows between the inner and outer electrodes of a capacitor, the equivalent permittivity varies with the water fraction, which in turn changes the measured capacitance [27]. In this study, a coaxial capacitive sensor is used to measure water holdup, and its configuration is schematically shown in Figure 2.

The sensor primarily consists of an inner electrode, an insulating layer, and an outer electrode. The key parameters are defined as follows: the sensor height is *H*; the central electrode radius is *r*; the radius of the inner electrode plus the insulating layer is *R*_1_; and the radius from the central axis to the outer electrode is *R*_2_. The annular region between the insulating coating and the outer electrode is filled with the measured oil–water mixture, forming the dielectric environment of the sensing field.

Let *ε_r_*_1_ denote the permittivity of the insulating layer and *ε_r_*_2_ denote the permittivity of the oil–water medium. If *Q* is the total charge carried by the inner electrode, the uniform surface distribution yields a linear charge density of *τ* = *Q*/*H*. For any point located at radial distance L from the central axis, the corresponding electric displacement vector is *D*, while *U* represents the potential difference between the two electrodes and *C_coa_* denotes the capacitance of the coaxial structure. By definition, the capacitance of the coaxial sensing unit can be written as

(3) $$C_{coa}=Q/U$$

For a cylindrical Gaussian surface of radius *L* and height *H*, Gauss’s law gives

(4) $$\oint_{s}Dds=Q=2\pi LDH$$

At this point, combined with the linear charge density, it follows that

(5) D=τ/2πL

Furthermore, by letting the electric field strength in the insulating layer be *E*_1_ and that in the measured oil–water mixture be *E*_2_, it follows that

(6) $$E_{1}=D/\left(\varepsilon_{0}\varepsilon_{r1}\right)=\tau /\left(2\pi L\varepsilon_{0}\varepsilon_{r1}\right)$$

(7) $$E_{2}=\tau /\left(2\pi L\varepsilon_{0}\varepsilon_{r2}\right)$$

where *ε*_0_ denotes the vacuum permittivity. Therefore, the capacitances of the insulating layer *C_εr_*_1_ and the measured oil–water mixture medium *C_εr_*_2_ can be expressed as follows:

(8) $$C_{\varepsilon r1}=2\pi \varepsilon_{r1}L/\left[\ln\left(R_{1}/r\right)\right]$$

(9) $$C_{\varepsilon r2}=2\pi \varepsilon_{r2}L/\left[\ln\left(R_{2}/R_{1}\right)\right]$$

Therefore, the potential difference *U* between the inner and outer electrodes and the capacitance *C_coa_* can be further expressed as follows:

(10) $$U=\int_{r}^{R_{2}}du=\int_{r}^{R_{1}}E_{1}dL+\int_{R_{1}}^{R_{2}}E_{2}dL=\frac{Q}{2\pi \varepsilon_{0}H}(\frac{1}{\varepsilon_{r1}}\ln\frac{R_{1}}{r}+\frac{1}{\varepsilon_{r2}}\ln\frac{R_{1}}{R_{2}})$$

(11) $$C_{coa}=C_{\varepsilon r1}\cdot C_{\varepsilon r2}/\left(C_{\varepsilon r1}+C_{\varepsilon r2}\right)=2\pi \varepsilon_{0}\varepsilon_{r1}\varepsilon_{r2}H/\left[\varepsilon_{r2}\ln\left(R_{1}/r\right)+\varepsilon_{r2}\ln\left(R_{2}/R_{1}\right)\right]$$

According to Equation (11), the capacitance value is related to the electrode dimensions of the sensor, the dielectric constant of the insulating material, and the equivalent permittivity of the measured medium. A larger sensor size results in a higher equivalent capacitance and thus improved measurement resolution; however, excessive dimensions are unfavorable for integration and restrict practical application scenarios. The measurement accuracy is mainly governed by the thickness of the insulating shell of the sensor. Provided that pressure resistance is ensured, a thinner insulating layer leads to higher measurement accuracy and sensitivity. The proposed sensor is applicable to bubbly flow, slug flow, and emulsified flow regimes.

Accordingly, when the sensor structure and insulating material are fixed, the capacitance of the capacitor depends on the equivalent permittivity of the oil–water mixture [28]. In practical measurements, an RC oscillation circuit is employed. Variations in water holdup lead to changes in the equivalent permittivity of the measured medium, resulting in different output frequencies of the RC oscillation circuit. By establishing the calibration relationship between the oscillation frequency and water holdup, the water holdup can be subsequently determined.

### 2.3. Principles of Flow Velocity Measurement

**Figure 3:**
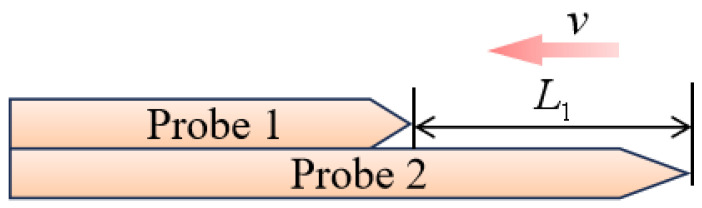
Schematic diagram of the fixed-spacing dual-probe measurement principle.

This study employs the fixed-spacing dual-probe measurement principle to determine flow velocity in downhole fluids, as illustrated in Figure 3.

Assuming the flow pattern remains unchanged during fluid movement, with a fixed distance *L*_1_ between the two probe tips, and denoting *t*_1_ as the time when the fluid contacts probe 2 and *t*_2_ as the time when it contacts probe 1, the flow velocity *v* of the downhole fluid is given by

(12) $$v=L_{1}/\left(t_{2}-t_{1}\right)$$

Considering that a gas bubble must successively contact both probes to enable effective gas flow measurement after reaching the first probe, an excessively large probe spacing reduces measurement effectiveness. Conversely, an overly small spacing degrades measurement resolution, and bubble deformation or turbulent flow may further increase measurement errors. These errors can be mitigated by averaging the measured signals. In practical engineering applications, probes with different spacings of *L*_1_ can be fabricated and selected according to downhole flow conditions, following the principle that the spacing of *L*_1_ should not be smaller than the bubble diameter. Optical-fiber probes are therefore suitable for gas holdup measurement in vertical wells with bubbly flow involving non-dissolved gas.

Based on the above analysis of the photoelectric composite three-phase flow sensor, it can be concluded that fiber-optic probes lose their resolution and measurement effectiveness in laminar and emulsion flows. In oil–water wells without gas, the sensor can be used for water holdup measurement in bubbly flow, slug flow, and emulsified flow. In gas wells without oil, it is applicable to gas holdup and flow rate measurements under bubbly and slug flow conditions. For oil–gas–water three-phase flows, the sensor is suitable for holdup and velocity measurements in vertical wells with bubbly flow.

Overall, the comprehensive performance of the sensor is closely related to a rational structural design. Therefore, the following section focuses on the structural design of the sensor, providing a foundation for subsequent experimental testing and result analysis.

## 3. Structural Design and Implementation of the Sensor

**Figure 4:**
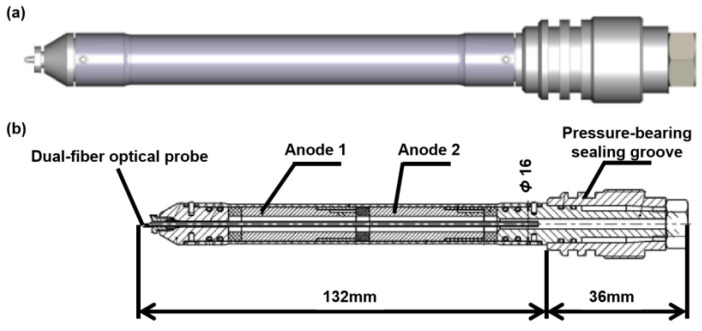
Configuration of the photoelectric composite three-phase flow sensor: (**a**) external layout; (**b**) internal cross-section showing probe and electrode assembly.

Based on the principles outlined in Section 2, the photoelectric composite three-phase sensor integrates tapered dual-fiber probes for gas holdup and flow-velocity measurement, together with a coaxial capacitive structure for downhole water holdup acquisition. Considering the bubble size in the wellbore, two probe tip distance *L*_1_ = 2 mm. Accordingly, the external structure and internal cross-section of the instrument are illustrated in Figure 4, highlighting the layout of fiber-optic probes and capacitive electrodes.

**Figure 5:**
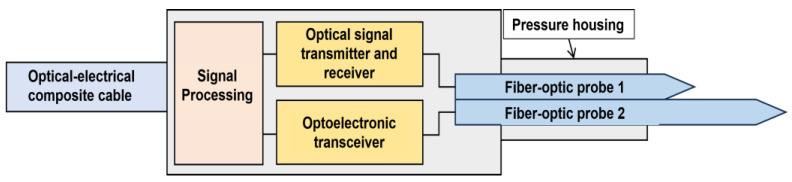
Schematic diagram of sensor signal reception.

As shown in Figure 4b, the tapered dual-fiber probes are placed at the front section of the housing, ensuring direct contact with the incoming flow for gas holdup and velocity detection. The rear section of the probes connects to an optical module for emission and detection, facilitating the generation and reception of optical signals. These signals are then transmitted to the signal processing module to complete gas holdup measurement. This process is schematically illustrated in Figure 5.

**Figure 6:**
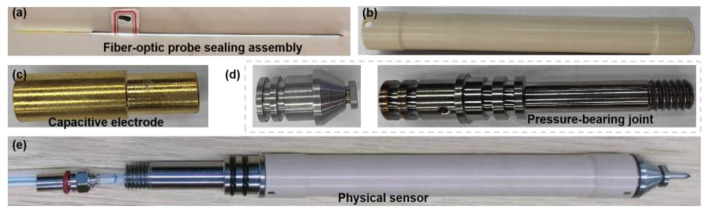
Components of the proposed three-phase flow sensor: (**a**) sealed fiber-optic probe assembly; (**b**) insulating housing; (**c**) capacitive electrodes; (**d**) pressure-bearing connectors; and (**e**) assembled sensor hardware.

Based on the aforementioned analysis, the designed photoelectric composite three-phase sensor and its component hardware are presented in Figure 6. As shown, the integration of pressure-bearing connectors and an insulating housing, containing sealed fiber-optic probes (incorporating optical-fiber sensors) and capacitive electrodes, enables accurate measurement of downhole fluid gas holdup, water holdup, and flow rates.

The sensing section has an outer diameter of 16 mm and a length of 132 mm. The mounting base is 36 mm in length and is designed with two sealing grooves and a threaded connection. Through the mounting base, the sensor can be securely installed onto the main body of the downhole measurement instrument.

## 4. Results and Analysis

### 4.1. Gas Holdup Measurement Results and Analysis

**Figure 7:**
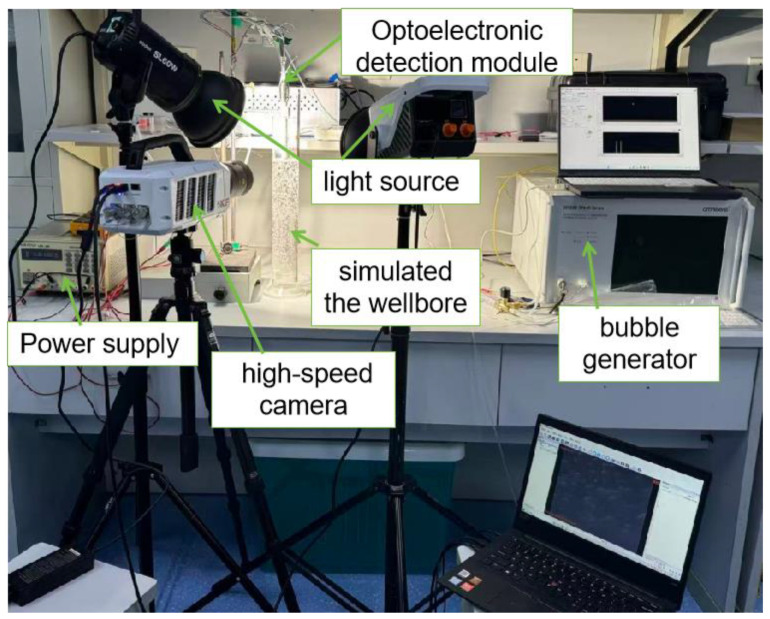
Hardware photograph of the gas holdup measurement system.

This study first evaluates the sensor performance with respect to gas holdup, and the experimental measurement system is shown in Figure 7. As illustrated, the system mainly consists of a power supply, oscilloscope, light source, photoelectric detection module, a vessel simulating the wellbore environment, and a bubble generator. For safety considerations, tap water, nitrogen, and diesel were selected as test media. The viscosity of diesel is 3.5 mPa·s and that of water is 1.00 mPa·s, and the bubble diameter ranges from 0.8 to 1.5 mm.

The power supply used in the experiment is a linear DC regulated power supply model LPS-305 (Ningbo Maodi Electronics Co., Ltd., Ningbo, China) with a maximum output power of 165 W, an output voltage range of 0–30 V, and an output current range of 0–2 A. The oscilloscope is an MSO2024 model (Tektronix, Beaverton, OR, USA) with a bandwidth of 200 MHz, four channels, a sampling rate of 1 GS/s, and a maximum waveform capture rate of 5000 wfm/s. A self-developed light source and photodetector are employed, with an optical power of 1 mW, a central wavelength of 980 nm, eight channels, a minimum detectable optical power of 10 μW, and an amplification factor of 10,000. A transparent acrylic cylinder is used to simulate the wellbore, allowing clear observation and recording of flow patterns. An oil-free air compressor model OTS-1-100X3 (Taizhou Aotusi Industry and Trade Co., Ltd., Taizhou, China) is adopted as the bubble generator, with a rotational speed of 1380 r/min, a nominal volumetric flow rate of 300 L/min, a rated exhaust pressure of 0.7 MPa, and an air storage tank volume of 100 L. A high-speed camera model 5f01 (Hefei Zhongke Junda Vision Technology Co., Ltd., Hefei, China) with a frame rate of 2000 FPS is used for flow visualization.

During the measurement process, the sensor is first placed in the gas–liquid two-phase flow vessel, and a certain amount of bubbles were generated by the bubble generator to form relative flow. When a gas bubble contacts the optical-fiber probe, total internal reflection occurs and the photodetector outputs a high-level signal. Using a high-speed data acquisition system, the response signals from the two probes and the corresponding timestamps are synchronously collected and stored. After each experimental run, the acquired data are plotted as two waveform sequences.

By analyzing the variation in the occurrence frequency of high-level signals in the two waveforms under different flow velocities, changes in gas holdup can be determined. Characteristic points with strong correlation between the two waveforms are identified, and, by comparing the corresponding timestamps with the recorded images, these signals are confirmed to be generated by the same bubble successively contacting the two probes. The gas bubble velocity is then calculated based on the time delay between the two waveforms.

**Figure 8:**
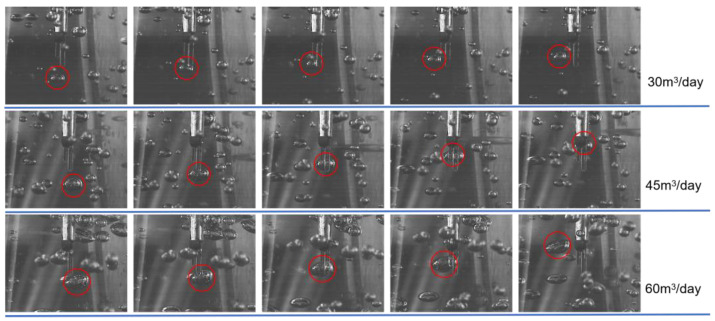
Bubble motion captured by high-speed camera at different gas velocities. The positions before and after the bubble contacts the probe are shown in red circles.

Furthermore, to accurately characterize the contact process between bubbles and the sensor probes and to determine the gas-phase flow velocity, the images captured by the high-speed camera at the instant of bubble–probe interaction under different flow conditions are carefully analyzed. The representative results are shown in Figure 8, where the circled regions indicate gas bubbles in the fluid.

**Figure 9:**
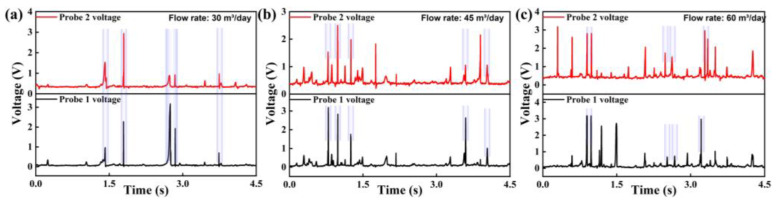
Variations in sensor output level under different gas velocities: (**a**) gas pump 30 m^3^/day, (**b**) gas pump 45 m^3^/day, and (**c**) gas pump 60 m^3^/day.The purple line indicates a correlation between the response waveforms of probe 1 and probe 2.

Based on the figure, it is observed that bubbles consistently contact the longer sensor probe first and subsequently slide towards the shorter section. This behavior suggests stable gas-phase movement, further supporting that the fluid flows within the container in a bubbly flow rather than a turbulent manner. Concurrently, as established in the preceding analysis, the sensor’s electrical level changes upon contact with a bubble. Accordingly, based on the observed bubble motion and its interaction pattern with the sensors, Figure 9 presents the variations in the sensor output level under different gas velocities.

Based on the figure, the sensor designed in this study accurately detects bubble contact. Simultaneously, as gas velocity increases, the number of high-level signals exhibits a gradual upward trend, indicating that higher velocities result in increased contact frequency between bubbles and the sensor probes. Furthermore, as established in the preceding analysis, the gas flow velocity can be calculated from the time difference between high-level signal occurrences on the two probes. Therefore, taking the gas pump 60 condition as an example, during the 13th bubble-contact event, Probe 1 recorded 0.95 V at 3.49 s while Probe 2 recorded 2.24 V at 3.50 s. Given the tip-to-tip distance between probes of *L*_1_ = 2 mm, the gas flow velocity is calculated as 0.2 m/s. When the flow rate is 60, the corresponding gas velocity is 0.185 m/s, from which it can be determined that the measurement error of the optical-fiber probe is 8.1%.

In summary, the photoelectric composite three-phase sensor developed in this study enables precise acquisition of gas holdup within oil well fluids, thereby providing a scientific basis for cost reduction and efficiency enhancement in petroleum extraction operations.

### 4.2. Water Holdup Measurement Results and Analysis

**Figure 10:**
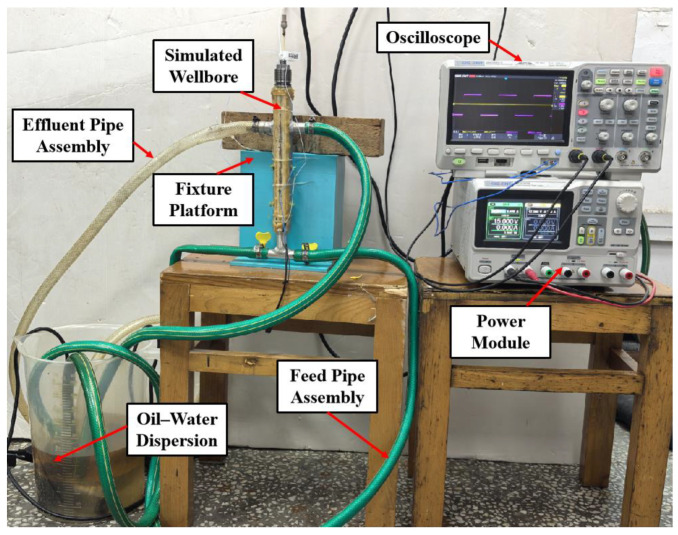
Hardware photograph of the water holdup measurement system.

To evaluate the detection performance of the sensor for water holdup, an experimental setup was designed as illustrated in Figure 10. The apparatus primarily consists of a power module, an oscilloscope, a fixture platform, feed and effluent pipes, and the multiphase flow sensor.

The fixture platform, fabricated via 3D printing, securely holds the simulated wellbore, within which the sensor is positioned to measure water holdup. A pump draws the oil–water mixture through the feed pipe into the simulated wellbore and subsequently discharges it via the effluent pipe back into the mixture reservoir, establishing a continuous flow. A standard flowmeter and pump are installed between the oil–water container and the detector experimental cylinder. The flow rate of oil, gas, and water is adjusted by the pump, and the flow rate displayed on the standard flowmeter is the standard flow rate.

**Figure 11:**
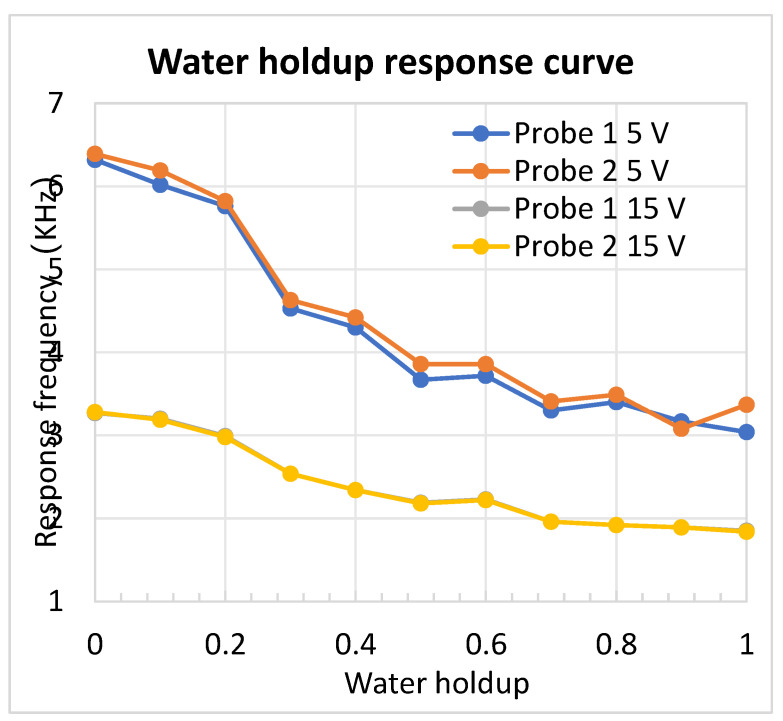
Water holdup measurement results.

A full-range calibration (0–100%, 10% increment) was conducted to evaluate sensitivity across the entire composition span, ensuring uniform resolution in both low- and high-water-cut regimes. The oil–water mixture was thoroughly homogenized using a constant-speed stirrer, and the water holdup was measured using the capacitance sensor, yielding the experimental results presented in Figure 11.

The figure indicates that under both 5 V and 15 V supply voltages, the trend of frequency variation with water holdup capacity is generally similar, except for the difference in frequency magnitude at an identical water holdup capacity. Specifically, within the 0% to 90% water holdup capacity range, the frequency value gradually decreases as the water holdup capacity increases. Moreover, in the low water holdup range (0–50%), the frequency amplitude decreases more rapidly, yielding a resolution as high as 1%. When the water holdup increases to 50–90%, the corresponding resolution is 3%. Comparing the measurement data with the calibration chart, the comprehensive measurement error is less than 3.4%. It is also noteworthy that at 100% water holdup capacity, the frequency value slightly increases compared to that at 90% (though it remains lower than values at other capacities). Additionally, comparing the frequency values measured by Probe 1 and Probe 2, under 5 V voltage, the measured values are identical except for slight differences observed at 40%, 50%, and 90% water holdup capacity, which might be attributed to local spatial variations in water holdup capacity at different sensor positions. Under 15 V voltage, the frequency values measured by Probe 1 and Probe 2 are the same.

Furthermore, studies indicate that water salinity exerts a measurable influence on capacitive-based water holdup measurements [5]. To evaluate salinity effects on the sensor developed in this study, a controlled variable approach was implemented wherein three sets of experiments with varying salinity levels were conducted at identical water holdup conditions. Salinity was adjusted by adding NaCl to the oil–water mixture. Taking 50% water holdup as an example, the impact of salinity on measurement outcomes is presented in Table 1.

Experimental results demonstrate that salinity exerts negligible influence on frequency measurements from both Probe 1 and Probe 2 under 5 V and 15 V supply voltages, indicating the sensor developed in this work exhibits strong immunity to adverse salinity effects in practical applications.

In summary, the photoelectric composite multiphase sensor developed in this study enables accurate determination of water holdup in downhole fluids while exhibiting enhanced resolution within the low-saturation regime.

### 4.3. Downhole Fluid Flow Rate Measurement Results and Analysis

**Figure 12:**
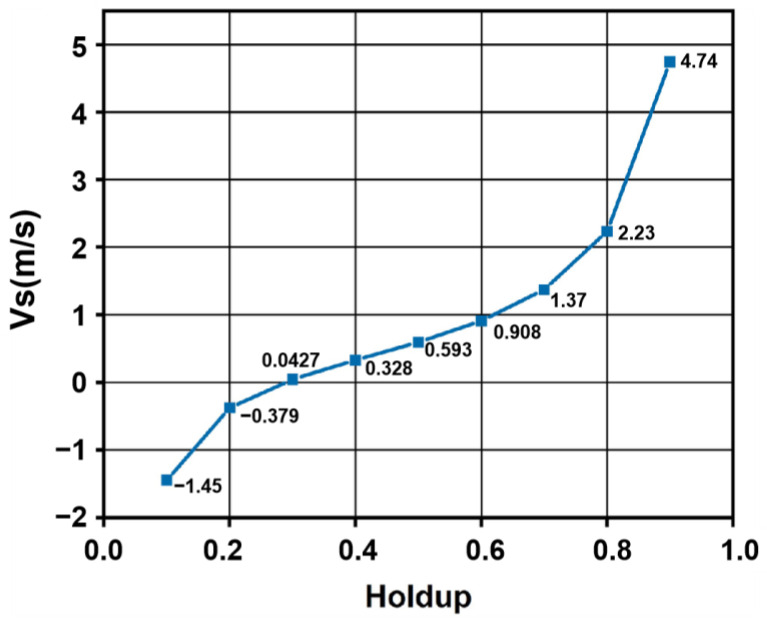
Variation in slip velocity with liquid holdup.

Further, in multiphase flow systems within petroleum engineering, inherent disparities in physical properties between phases and interfacial interactions induce velocity differences in the actual mean velocities of distinct phases (e.g., gas and liquid). This differential velocity, defined as the slip velocity *V_s_*, exhibits a positive correlation with the degree of flow non-uniformity, as quantified by Equation (13):

(13) $$V_{s}=\frac{V_{g}}{A_{w}H_{f}}-\frac{V_{f}}{A_{w}(1-H_{f})}$$

where *V*_g_ and *V_f_* represented the gas- and liquid-phase volumetric flow rates, respectively, Aw denotes the pipe cross-sectional area, and *H_f_* is the liquid holdup. This paper investigates the variation in gas–liquid slip velocity with liquid holdup under constant volumetric flow rate conditions, as shown in Figure 12.

As illustrated in Figure 12, the slip velocity increases with increasing liquid holdup, indicating a corresponding increase in flow heterogeneity between the gas and liquid phases. Specifically, at low liquid holdup levels (10% and 20%), the slip velocity exhibits negative values, signifying that the actual gas velocity is lower than the actual liquid velocity. This suggests a potential risk of gas accumulation under these conditions. Conversely, within the liquid holdup range of 30% to 90%, the slip velocity becomes positive, reflecting a higher actual gas velocity relative to the liquid velocity. This occurs because higher liquid holdup reduces the available gas flow area, leading to constriction-induced acceleration of the gas phase and consequently greater gas velocity compared to liquid velocity.

After the holdup is obtained, the liquid-phase velocity is calculated using the slip model. In this study, the total oil–water holdup of the test sample is 80%, and the slip velocity is *v*_*s*_ = 2.23 m/s. For a typical oilfield casing with an inner diameter of 125.7 mm and a measured gas velocity of *v*_*g*_ = 0.2 m/s, substitution into Equation (13) yields a liquid-phase velocity of 0.04 m/s. Neglecting the measurement error of the gas-phase velocity, the actual liquid flow rate is converted to a velocity of 0.043 m/s based on the experimental pipe diameter, resulting in a measurement error of 6.9%.

To further evaluate the performance of the proposed sensor, the experimental results are compared with sensor specifications reported in the literature. The key performance metrics are summarized in Table 2.

It should be noted that the electrode insulation layer of the sensor is made of PEEK material, which exhibits good thermal and pressure resistance but has a certain degree of water absorption. When exposed to an oil–water environment for extended periods, water absorption may alter the effective dielectric constant of the insulation layer, leading to drift in the sensor response. In addition, when an oil film forms on the surface of the optical-fiber probe, the refractive index of the sensitive region may change, thereby reducing the effective detection capability of the sensor. In such cases, adjustment of the light source wavelength may be required. These two effects have not yet been experimentally verified and therefore constitute sources of uncertainty in the present measurement system.

### 4.4. High-Temperature Validation of Sensor Performance

**Figure 13:**
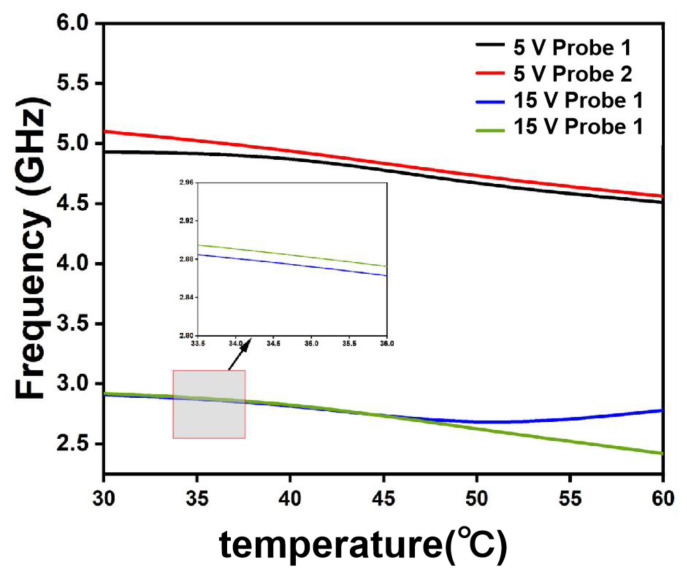
Influence of temperature on water holdup.

As indicated by the foregoing analysis, the measurement principle of water holdup relies on the difference in dielectric constants between oil and water. However, at elevated temperatures, the dielectric constant of water decreases significantly with increasing temperature. Furthermore, high temperatures reduce the viscosity of crude oil and alter the oil–water mixing state, thereby further affecting the accuracy of the measurement results. Therefore, to investigate the influence of high-temperature conditions on water holdup measurement, a controlled variable approach was employed under a fixed water holdup of 50%. The measured water holdup values at different temperatures are presented in Figure 13.

As observed in the figure, a comparison with the slopes of the curves under the 5 V supply reveals that the slopes of the curves under the 15 V supply decrease slightly more markedly. Moreover, the slope decrease for Probe 2 is marginally greater than that for Probe 1, indicating that the probe frequency exhibits greater sensitivity to temperature under the 15 V power supply, alongside minor differences between the probes. Nevertheless, the overall decline of all curves remains gradual with increasing temperature, implying that the influence of temperature on the measurements from both Probe 1 and Probe 2 is limited under both the 5 V and 15 V power supply conditions. This further confirms that the sensor developed in this work demonstrates robust resistance against the adverse effects of high temperature on measurement performance in practical applications.

## 5. Conclusions

Regarding the measurement of logging parameters, including water holdup, gas holdup, and fluid flow rate, this study employs a dual-fiber optical probe and has conducted the following work:

(1) Based on the principle of total reflection, the gas holdup was measured using the fiber-optic probe method.
(2) The coaxial capacitive sensor was employed to measure the fluid water holdup, and the influence of water salinity on the measurement results was also discussed.
(3) Based on the liquid flow rate obtained from the dual optical-fiber probes, a gas–liquid slip velocity model was introduced. The variation in gas–liquid slip velocity with water holdup was analyzed, and the liquid-phase velocity was calculated using the proposed model.
(4) High-temperature experiments were conducted on the sensor to evaluate the impact of temperature on its water holdup measurement performance.

In summary, the experimental results demonstrate that the proposed photoelectric composite three-phase flow sensor can accurately measure gas holdup, water holdup, and gas–liquid two-phase flow velocity. Moreover, it shows good robustness against the effects of salinity and temperature on measurement accuracy in practical applications. Featuring high measurement accuracy, a simple structure, and a high level of integration, the sensor will be further investigated in oil–gas–water three-phase flow facilities. After a comprehensive understanding of its response characteristics is achieved, its limitations and applicability will be clarified, indicating strong potential for large-scale application in dynamic monitoring of complex three-phase flow wells.

## Figures and Tables

**Table 1:** Effects of salinity on water holdup measurements.

Mass of NaCl Added (g)	5 V Power Supply		15 V Power Supply	
	Probe 1 Frequency (kHz)	Probe 2 Frequency (kHz)	Probe 1 Frequency (kHz)	Probe 2 Frequency (kHz)
0	3.37	3.86	2.19	2.18
150	3.20	3.37	2.15	2.16
300	3.18	4.01	2.00	2.38
500	3.21	4.05	2.06	2.52

**Table 2:** Comparison of key performance parameters of different sensors.

Sensor	Sensing Principle	Gas Holdup	Water Holdup	Phase Velocity	Integration Level	Typical Resolution
Capacitance [20]	Capacitance	No	Yes	No	Single-parameter	<1% (water holdup)
Microwave resonant [21]	Microwave	No	Yes	No	Single-parameter	≈3% (high water cut)
Fiber-optic [23]	Photoelectric	Yes	Yes	No	Single-function array	~10% (phase holdup)
Conductance [17]	Mechanical + Electrical	No	Yes	Liquid only	Multi-module tool	~10% (oil–water)
This work	Photoelectric + Capacitance	Yes	Yes	Gas and liquid	Highly integrated	1–3% (water holdup)

## Data Availability

The data supporting the findings of this study are available from the corresponding author upon reasonable request.
